# Phylogenetic Analysis of Hepatitis C Virus Infections in a Large Belgian Cohort Using Next-Generation Sequencing of Full-Length Genomes

**DOI:** 10.3390/v15122391

**Published:** 2023-12-08

**Authors:** Kasper T. Christensen, Florian Pierard, David Bonsall, Rory Bowden, Eleanor Barnes, Eric Florence, M. Azim Ansari, Dung Nguyen, Mariateresa de Cesare, Frederik Nevens, Geert Robaeys, Yoeri Schrooten, Dana Busschots, Peter Simmonds, Anne-Mieke Vandamme, Eric Van Wijngaerden, Tim Dierckx, Lize Cuypers, Kristel Van Laethem

**Affiliations:** 1 Laboratory of Clinical and Epidemiological Virology, Department of Microbiology, Immunology and Transplantation, Rega Institute for Medical Research, KU Leuven, 3000 Leuven, Belgium; florian.pierard@kuleuven.be (F.P.); yoeri.schrooten@uzleuven.be (Y.S.); annemie.vandamme@kuleuven.be (A.-M.V.); tim.dierckx@kuleuven.be (T.D.); lize.cuypers@uzleuven.be (L.C.); kristel.vanlaethem@uzleuven.be (K.V.L.); 2 Big Data Institute, Li Ka Shing Centre for Health Information and Discovery, Nuffield Department of Medicine, University of Oxford, Oxford OX3 7LF, UK; david.bonsall@bdi.ox.ac.uk; 3 The Wellcome Centre for Human Genetics, University of Oxford, Roosevelt Drive, Oxford OX3 7BN, UK; bowden.r@wehi.edu.au (R.B.); dung.nguyen@ndm.ox.ac.uk (D.N.); mariateresa.decesare@fht.org (M.d.C.); 4 Peter Medawar Building for Pathogen Research, University of Oxford, Oxford OX1 3SY, UK; ellie.barnes@ndm.ox.ac.uk; 5 Translational Gastroenterology Unit, University of Oxford, Oxford OX3 9DU, UK; 6 Oxford NIHR Biomedical Research Centre, University of Oxford, Oxford OX3 9DU, UK; 7 Department of General Internal Medicine, Infectious Diseases and Tropical Medicine, Antwerp University Hospital, 2650 Edegem, Belgium; eric.florence@uza.be; 8 Department of Clinical Sciences, Institute of Tropical Medicine, 2000 Antwerp, Belgium; 9 Nuffield Department of Medicine, University of Oxford, Oxford OX3 7BN, UK; azim.ansari@ndm.ox.ac.uk; 10 Department of Gastroenterology and Hepatology, University Hospitals Leuven, 3000 Leuven, Belgium; frederik.nevens@uzleuven.be (F.N.); geert.robaeys@uhasselt.be (G.R.); 11 Faculty of Medicine and Life Sciences—LCRC, UHasselt, Agoralaan, 3590 Diepenbeek, Belgium; danabusschots@hotmail.com; 12 Department of Gastroenterology and Hepatology, Ziekenhuis Oost-Limburg, 3600 Genk, Belgium; 13 Department of Laboratory Medicine, University Hospitals Leuven, 3000 Leuven, Belgium; 14 Henry Wellcome Building for Molecular Physiology, Nuffield Department of Medicine, University of Oxford, Old Road Campus, Headington, Oxford OX3 7BN, UK; peter.simmonds@ndm.ox.ac.uk; 15 Global Health and Tropical Medicine, Institute of Hygiene and Tropical Medicine, Universidade Nova de Lisboa, Rua da Junqueira 100, 1349-008 Lisbon, Portugal; 16 Department of General Internal Medicine, University Hospitals Leuven, 3000 Leuven, Belgium; eric.vanwijngaerden@uzleuven.be; 17 Laboratory of Clinical Microbiology, Department of Microbiology, Immunology and Transplantation, KU Leuven, 3000 Leuven, Belgium

**Keywords:** hepatitis c, human immunodeficiency virus, phylogenetic analysis, full-genome sequencing, men who have sex with men, people who inject drugs, next-generation sequencing

## Abstract

The hepatitis C virus (HCV) epidemic in Western countries is primarily perpetuated by the sub-populations of men who have sex with men (MSM) and people who inject drugs (PWID). Understanding the dynamics of transmission in these communities is crucial for removing the remaining hurdles towards HCV elimination. We sequenced 269 annotated HCV plasma samples using probe enrichment and next-generation sequencing, obtaining 224 open reading frames of HCV (OR497849-OR498072). Maximum likelihood phylogenies were generated on the four most prevalent subtypes in this study (HCV1a, 1b, 3a, 4d) with a subsequent transmission cluster analysis. The highest rate of clustering was observed for HCV4d samples (13/17 (76.47%)). The second highest rate of clustering was observed in HCV1a samples (42/78 (53.85%)) with significant association with HIV-positive MSM. HCV1b and HCV3a had very low rates of clustering (2/83 (2.41%) and (0/29)). The spread of the prevalent subtype HCV1b appears to have been largely curtailed, and we demonstrate the onwards transmission of HCV1a and HCV4d in the HIV-positive MSM population across municipal borders. More systematic data collection and sequencing is needed to allow a better understanding of the HCV transmission among the community of PWID and overcome the remaining barriers for HCV elimination in Belgium.

## 1. Introduction

Globally, 58 million people suffer from chronic hepatitis C virus (HCV) infections, with an estimated 1.5 million new infections and 290,000 deaths every year [1]. As a blood-borne pathogen, infected blood products were a major contributor to the spread of HCV before antibody screening tests were developed in 1989 [2]. While iatrogenic infections remain a significant route of transmission in resource-limited settings, the HCV epidemics of Western countries are now mainly perpetuated in defined subpopulations such as the incarcerated, people who inject drugs (PWID), and men who have sex with men (MSM). HCV and human immunodeficiency virus (HIV) share similar socio-demographic risk factors and transmission routes, and a co-infection can complicate and accelerate the disease progression of HCV [3,4,5]. Treatment as prevention is the most important tool for managing the epidemic outside of harm reduction initiatives such as needle exchange programmes, since modern direct-acting antivirals (DAA) can cure >95% of treated HCV infected patients [6,7], and no prophylactic vaccine is available. With HCV primarily spread among specific key populations in Western countries, focusing resources on HCV management in micro-eliminations of at-risk communities has become the target of intense study [8,9,10,11]. The success of these strategies, however, depends heavily on the veracity and resolution of epidemiological data [12].

Viral sequence data can be used to infer the nature of transmission networks and the momentum with which the active clusters sustain the HCV epidemic. Risk factors associated with ongoing transmission as well as the potential spill-over between key populations can be defined when the viral sequence data is annotated with these metadata [13,14,15,16,17,18]. However, obtaining HCV sequencing data is challenging due to the high genetic variability of HCV with more than 90 recognized subtypes distributed across eight genotypes [19]. As the nucleotide diversity is unevenly distributed across the HCV genome [20], the choice of genomic region used in phylogenetic analyses can heavily influence the final topology of the phylogenetic tree with longer sequences generally considered favourable [21,22]. Full-genome sequencing of HCV, regardless of sub- and genotype, has only recently become practically feasible [23,24], but thus far, phylogenetic studies of HCV using full genomes have been limited in either cohort size or subtype distribution [25,26,27] with a few notable exceptions [28,29,30].

In this study, we included the samples and data of 267 HCV patients from different key populations who attended three centres providing HIV and HCV clinical care in the north-eastern region of Belgium and investigated risk factors associated with onwards transmission through phylogenetic clustering of HCV open reading frames (ORF).

## 2. Materials and Methods

### 2.1. Patients

Blood plasma samples were collected as part of routine clinical care for the assessment of HCV viral load between 2006 and 2018 from patients attending three clinical centres in the north-eastern region of Belgium within the context of a study focusing on populations at increased risk of HCV transmission and HIV coinfected patients. Samples were annotated with the available socio-demographic and laboratory information (year of birth, country or region of origin, gender, year of HCV diagnosis, sexual orientation, HIV co-infection status, history of intravenous drug use (IVDU), and other risk factors). Iatrogenic infections were defined as infections acquired by haemophilia patients, or by people who declared a professional risk or a transfusion as a risk factor. Migrants were defined as people who reported a country of origin other than Belgium. The UZ Leuven hospital contributed 138 patients, the Institute of Tropical Medicine Antwerp (ITM) contributed 53 patients, and Ziekenhuis Oost-Limburg (ZOL) contributed 76 patients. For each patient, only one sample was included, unless there was evidence of a re-infection. This study was approved by the Ethical Committee Research UZ/KU Leuven (S61339).

### 2.2. Sequences

The samples were sequenced in seven batches using variations on the ve-SEQ protocol [31] with the samples from Leuven obtained using the unaltered version. In short, samples were extracted on a NucliSENS easyMAG (bioMérieux, Marcy-l’Étoile, France). Libraries were prepared using NEBNext Ultra II Directional RNA Library Prep Kit for Illumina (New England BioLabs, Ipswich, MA, USA), enriched using an aliquot of the probe panel designed by the developers of the ve-SEQ protocol, and sequenced on an Illumina MiSeq using 150 cycle paired-end reads. The libraries from Antwerp and Genk were prepared using a slight variation on the ve-SEQ protocol with adaptations for higher depths of coverage and longer inserts. Briefly, samples were extracted instead using the QIAamp Viral RNA Mini Kit (QIAGEN, Hilden, Germany) using carrier RNA, DNase, and the RNeasy MinElute Cleanup Kit (QIAGEN). The initial steps of the NEBNext Ultra II Directional RNA Library Prep Kit for Illumina were modified to use instead SuperScript IV rather than the reverse transcriptase native to the kit with a decreased fragmentation time of five minutes. The second strand synthesis was increased to 180 min, the volume of beads in the fragment size selection step was decreased from 0.9× to 0.6×, and samples with a viral load below the 1st and 2nd quartile for all samples in a pool had their calculated volumes for equimolar pooling modified by a factor of four and two, respectively, to compensate for the expected lower proportion of fragments of HCV origin. Finally, the number of cycles in the PCR enrichment of the pooled library was increased to twelve, and the number of sequencing cycles was increased to 250.

Paired-end reads were processed bioinformatically using an in-house pipeline that cleans and trims reads using TRIMMOMATIC [32] and Trim Galore [33] before de novo analysis using both IVA [34] and SPAdes [35]. A sample-specific reference was generated using the assembled HCV contigs with contigs flagged as contaminants using BLAST removed with shiver [36]. Finally, reads were mapped using BWA-MEM [37], and a cut-off of 15% was used for calling nucleotide ambiguity codes.

### 2.3. Phylogenetic Construction

For a sequence obtained through NGS to qualify for phylogenetic analysis, we set out two criteria that had to be met. First, >90% of the ORF had to be covered to a depth of >30 reads/site. Second, there had to be an agreement in subtype assignments of the cohort sequence using a local BLAST of the ORF against 243 curated subtype references downloaded from the International Committee for the Taxonomy of Viruses and using Genome Detective. Details on the qualifying sequences are outlined in Table 1, and a phylogenetic tree can be found in Supplementary Figure S1.

**Figure 1:**
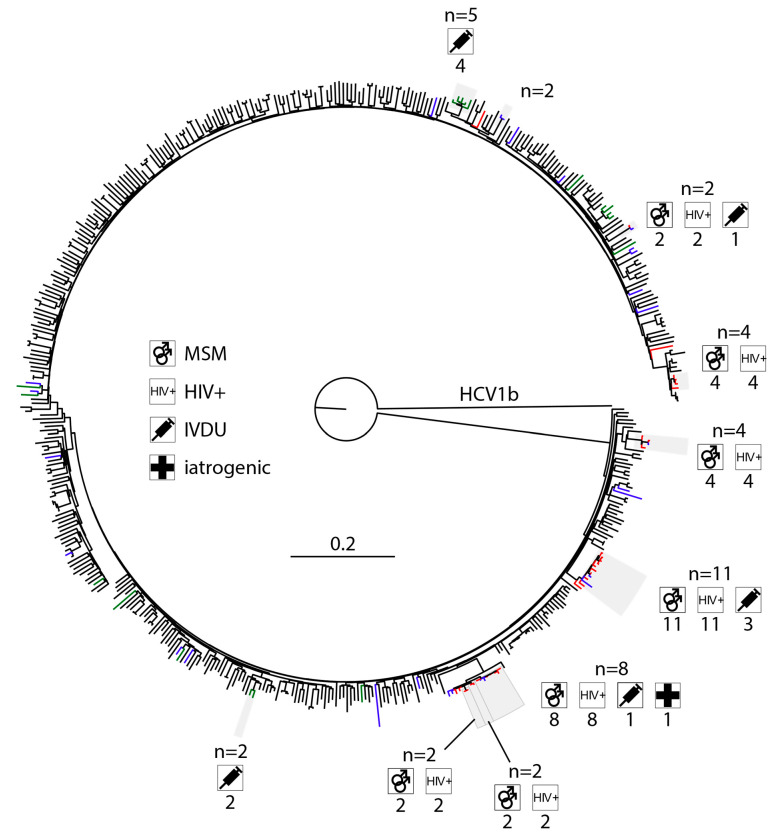
A maximum-likelihood tree was constructed based on HCV1a ORFs using MAFFT and IQ-Tree 2 with automatic substitution model detection (TVM + F + R10) and 1000 ultra-fast bootstrap replicates and was rooted using an HCV1b outgroup. Blue, red, and green branches correspond to samples originating from Leuven, Antwerp, and Genk, respectively. Clusters based on bootstrap and genetic distance thresholds of 90% and 4 and are highlighted in grey. Pictograms indicate the characteristics of each cluster.

**Figure 2:**
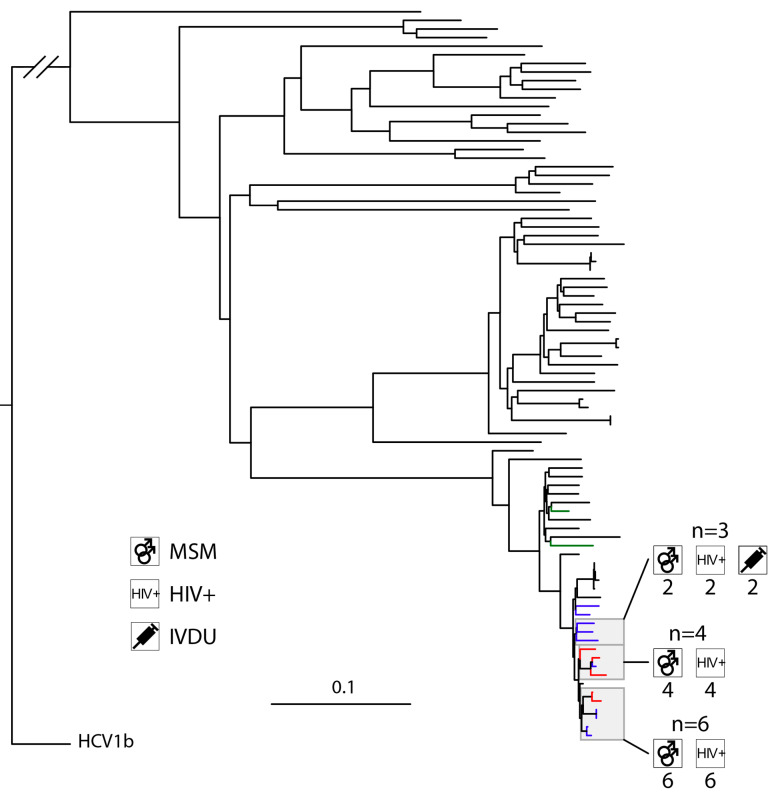
A maximum-likelihood tree was constructed based on HCV4d ORFs using MAFFT and IQ-Tree 2 with automatic substitution model detection (TVM + F + R10) and 1000 ultra-fast bootstrap replicates and was rooted using a HCV1b outgroup. Blue, red, and green branches correspond to samples originating from Leuven, Antwerp, and Genk, respectively. Clusters based on bootstrap and genetic distance thresholds of 90% and 4 and are highlighted in grey. Pictograms indicate the characteristics of each cluster.

To collect appropriate control sequences, a local database of 20,093 publicly available sequences was generated from all GenBank sequences classified as Hepacivirus C (txid11103) of between 2500 and 10,000 nucleotides in length from the National Center for Biotechnology Information, consulted on 11/04/2023. All HCV sequence records annotated with variations of the phrase “cell culture” or had a host other than “Homo sapiens” were removed. The qualifying sequences were split into sets of subtypes, and the closest 50 hits for each cohort sequence within a set was determined using BLAST against the local database. An in-house Python script removed control sequences with identical accession numbers as well as distinct accession numbers with identical sequences retaining the record with most annotations (place and time of sampling) where possible. The remaining BLAST hits were written to a fasta file together with their cohort sequences and aligned using MAFFT [38]. Alignments were manually checked and trimmed if necessary and a first phylogeny generated using FastTree. The resulting trees were reviewed, and large clades of very related non-cohort sequences manually reduced to one representative sequence before realignment using MAFFT (--maxiterate 1000 --globalpair) for the final phylogeny with IQ-Tree 2 [39] using 1000 ultra-fast bootstraps and automatic substitution model detection (Figure 1 and Figure 2 and Supplementary Figures S2 and S3). The selected substitution models were TVM + F + R10, TIM2 + F + R10, GTR + F + R6, and TVM + F + R10 for HCV1a, 1b, 3a, and 4d, respectively. Quartet puzzling with the maximum likelihood criterion in TREE-PUZZLE was used to assess the percentage of conflicting phylogenetic signals [40].

### 2.4. Transmission Cluster Analysis

We used Cluster Picker to identify clusters using the common cut-off for genetic distances of 4% [14,15,41] with a minimum bootstrap support threshold of 90%, but we performed a sensitivity analysis using a range of genetic distances between 2% and 8% (see Table 2). As we are interested in onwards local transmission, only initial clusters with two or more sequences from the cohort described here were considered for cluster transmission analysis. We performed a Fischer’s exact test to investigate the association of individual socio-demographic parameters to transmission within a cluster with a Bonferroni-corrected α  =  0.05/4  =  0.0125 significance level for reported odds ratios.

## 3. Results

### 3.1. Participant Characteristics

We included 269 blood plasma samples and their clinical annotation from 267 patients (one reinfection and one multiple infection). The different centres exhibited different profiles in their individual cohorts (see Table 1) with higher relative frequencies of male patients, same-gender sexual attraction, and HIV-positive status among persons attending ITM (97.6%, 95.2%, and 100%), PWID and European migrants in persons attending ZOL (31.0% and 33.8%), and iatrogenic infections in the cohort of Leuven patients (33.3%). Similarly, the distributions of subtypes of HCV were distinct with comparatively higher levels of HCV1a and 4d in persons attending ITM and higher levels of HCV3a in the cohort from ZOL. The three cohorts also exhibited differences in age and treatment history with a nine-, ten-, and five-year gap between the oldest and most recent median year of birth, diagnosis, and sampling.

### 3.2. Sequencing Results

From Leuven, Antwerp, and Genk, 111, 42, and 71 sequences passed the criteria on subtype classification and width- and breadth of sequencing, respectively. Among the 224 HCV ORFs, HCV1a (78, 34.8%), 1b (83, 37.1%), 3a (29, 12.9%), and 4d (17, 7.6%) were the major subtypes within the entire cohort and were further subjected to individual phylogenetic and transmission cluster analyses. The remaining 17/224 ORFs were four sequences of subtype HCV4k, two each of subtype HCV2c, 4a, and 4r, and one each of subtypes HCV1c, 2a, 2i, 4c, 4q, and 4v. The last remaining ORF was not identified using Genome Detective but was classified as a HCV2k1b recombinant after a more extensive analysis [42]. As expected for full ORFs, the suitability of our sequence data for phylogenetic analysis was very high: all sequences had percentages of unresolved quartets below 0.1% according to TREE-PUZZLE, indicating a harmonious phylogenetic signal. The consensus sequences of the 224 ORFs identified here has been uploaded to GenBank using a 15% threshold for calling IUPAC ambiguity code (accession numbers: OR497849-OR498072).

### 3.3. High Levels of Clustering in HIV-Positive MSM with HCV1a and 4d Infections

Only subtypes HCV1a and 4d had more than one transmission cluster, defined at a bootstrap >90% and at a genetic distance threshold of 4. The rate of clustering was 53.85% in HCV1a and 76.47% in HCV4d. The size of the ten transmission clusters identified in HCV1a was 11, 8, 5, 4, and 4 with a further five transmission pairs. HCV4d had three clusters of sizes 6, 4, and 3. For HCV1b, only a single cluster with two sequences was identified (2.41%). Subtype HCV3a had no clusters at a genetic distance threshold of 4. Clustering in HCV1a was associated with HIV positive MSM (OR 18.33). Country of origin other than Belgium at a genetic distance of 4 had a *p*-value of 0.03, which does not meet the Bonferroni-adjusted threshold for statistical significance. At a genetic distance of 6 and 8, however, the inverse correlation between migrant status and clustering was found to be statistically significant.

Across all genetic distances in the sensitivity analysis, no parameter achieved a statistically significant correlation to clustering for subtypes HCV1b, 3a, and 4d. Only 1/32 (3.13%) of the HCV1a infected patients with a potential iatrogenic infection was situated within a cluster but this sample originated from a patient belonging to both the PWID and MSM communities in addition to suffering from haemophilia and remained the only potentially iatrogenic infection in a HCV1a cluster throughout the sensitivity analysis.

## 4. Discussion

We obtained 224 HCV ORFs comprising a diverse set of 15 total observed HCV subtypes and recombinants. To our knowledge, the research presented here represents the largest phylogenetic study of HCV using full ORFs to date. Similarly, there have been phylogenetic studies of HCV sequences from Belgium specifically before, but previous studies were performed in smaller cohorts, on individual subtypes, on smaller fragments, or without an analysis of transmission pathways [43,44,45,46].

### 4.1. The Spread of HCV1b Has Largely Been Suppressed

While the size of our cohort is considerable, the long sampling interval of 12 years across multiple clinical centres and risk groups means that while our cohort is large, it may be less densely sampled than other studies featuring a smaller but more focused cohort. This is likely part of the explanation for the relatively small rate of clustering identified for some of the subtypes and risk groups of this study: we have only obtained very few representative sequences from much larger, broken transmission chains. Whereas this may present as a shortcoming for this study to describe any one subpopulation in detail, it provides a novel perspective on broader dynamic trends of persistent strains—especially if a higher tolerance of cluster definitions is permitted to accommodate the change in resolution. However, even at a genetic distance threshold of 6%, only 6/83 (7.23%) HCV1b sequences were part of a cluster—all of which were transmission pairs. Two of these transmission pairs had patients diagnosed around the turn of the millennium (1995 and 2001, 1998 and 2002) with the most recent pair being a vertical transmission diagnosed in 2013 and a 2008 diagnosis recorded as accidental. All this seems to indicate that HCV1b may be losing its momentum in Belgium. This is backed up by the discrepancy in diagnosis and sampling times across the four major subtypes. While the median years of sampling are relatively similar, being 2014, 2015, 2015, and 2013 for subtypes HCV1a, 1b, 3a, and 4d, the median years of diagnosis are 2009, 2004, 2009, and 2011. Taken together with the phylogenetic analysis, this suggests that despite being a major subtype within the population (and the largest subtype in our cohort), the contemporary Belgian HCV1b infection is less likely to lead to onward transmission than HCV1a or 4d. This is likely in part due to HCV1b’s high rate of iatrogenic infections and low frequency of PWID and HIV-positive MSM (see Table 3), but the scarce information on the Belgian epidemic does suggest that iatrogenic infections could be underrepresented in our cohort [43]. 

### 4.2. HCV3a Is Highly Prevalent in an Undersampled PWID Community

Even at a genetic distance threshold of 6%, only 3/29 (10.34%) of HCV3a sequences clustered. Despite a similar rate of clustering to HCV1b, the characteristics of the patients infected with these subtypes are very distinct, with HCV3a exhibiting the highest proportion of PWID among all the major subtypes. A systematic review from 2014 found HCV3a to be one of the main subtypes circulating in PWID in Europe [44], with two earlier studies identifying this subtype as the most frequent subtype among PWID in Belgium specifically with a prevalence of 41.2% and 49% [45,46]. The single cluster identified at a genetic distance of 6% consisted of three patients from Genk within a larger clade of six sequences only from Genk. Since PWID are a major contributor to the epidemic in Belgium, this close association between sequences that still do not cluster can be seen as evidence of the existence of a large network of PWID with unsampled transmission chains. This is supported by HCV3a exhibiting the largest proportion of both samples from patients reporting a country of origin outside Belgium and IVDU. Additionally, two of the sequences in the cluster were PWID with Turkey as their country of origin, where HCV3a is the predominant genotype found in PWID [47].

### 4.3. HCV Networks in Heterosexual PWID and MSM PWID Appear to Be Distinct

Across the four major subtypes at a genetic distance of 4%, 4/14 (28.57%) clusters simultaneously contained both PWID and MSM but only because of patients who fulfilled both criteria. Thus, there were no clusters that contained both patients recorded only as PWID and other patients recorded only as MSM. Only at a genetic distance of 8% did a single HCV1a cluster appear that contained both patients who were PWID but not MSM and patients who were MSM but not PWID. Therefore, while there is some potential for PWID who are also MSM to bridge the gap between the two communities, we found no clear evidence of a substantial overlap between the strains circulating in the discrete subpopulations. It is possible that the community of PWID can be further broken down into two distinct groups that interact very little: i.e., separate heterosexual PWID and MSM PWID communities. A social separation of the two would be congruent with the epidemiological disconnect hinted at in our cohort, despite their surface-level behavioural similarities. This potential aspect of the epidemic is supported by previous studies indicating that heterosexual men and MSM exhibit discrete patterns in the specific intravenous drugs used by each subpopulation [48,49] and their reason for injecting [50]. 

### 4.4. HCV4d Has Seen Substantial Transmission in HIV-Positive MSM

A combined keyword search on GenBank and BLAST search for similarity to our 4d sequences suggests that the platform only has 16 non-clonal full ORFs as of our dataset pulled 11/04/23. Here, we obtained a further 17 ORFs of subtype HCV4d significantly increasing the number of sequences publicly available on GenBank. Previous works have found connections between the HCV4d transmission networks in MSM among several European countries as well as Australia and Canada, with one study finding that spread could not be attributed to IVDU [30,51,52,53]. Of our 17 HCV4d sequences, 13 were in a cluster, 12 of which were HIV-positive MSM. In our cohort, HCV4d had the most recent median year of diagnosis (2011) and the highest rate of clustering (76.47%), indicating that HCV4d has been rapidly spreading in HIV-positive MSM. Interestingly, in one cluster, all three sequences were from patients with three distinct foreign origins (the Netherlands, Poland, Ethiopia) suggesting a large but potentially undersampled international epidemic. Despite this, there was no statistically significant correlation between migrants and clustering status at a genetic distance threshold of 4 for HCV4d. For HCV1a, however, we observed a statistically significant inverse correlation between migrants and belonging to a cluster for genetic distances 6 and 8. It appears that for HCV1a specifically, which could soon be the most prevalent subtype in Belgium considering the apparent decline of HCV1b, migrants do not significantly contribute to onwards transmission.

### 4.5. HIV-Positive MSM Transmit HCV across Municipal Borders

Across the 14 clusters identified for all four subtypes tested, six clusters contained patients from a mix of clinical centres, all of which were Antwerp and Leuven. All of the 35 patients in these mixed clusters were HIV-positive MSM. No clusters with Genk samples contained samples from any other clinical centre. While this dataset contains both HCV patients with and without an HIV infection, we were unable to perform a phylogenetic analysis on the HIV infections of the same patients due to insufficient sample volume, and as such, we are unable to contrast the networks to investigate a potential overlap of the two epidemics. However, only a single cluster contained both patients with and without HIV infections. This was a three-person HCV4d cluster with two international HIV-positive MSM, together with a Polish man with no recorded transmission risks. The relative rarity of mixes in the HIV co-infection status of our clusters could be another effect of the potential bias of our cohort towards a dense network of HIV-positive MSM and relative under-sampling of the PWID community, but it is supported by previous research indicating that European HIV-positive MSM are spreading HCV in international transmission networks distinct from those of PWID [52]. While the overlap in the transmission chains of HCV and HIV in MSM is disputed [54,55,56], there is evidence to suggest that the networks of HCV and HIV in PWID is rather high, with 59.8% of patients of a Romanian cohort sharing at least one other patient in both their HCV and HIV clusters [57].

### 4.6. Study Limitations

Our study has a few limitations. First, as very few sequences of subtype HCV4d are publicly available, and only 17 sequences of this subtype are in the cohort presented here, the phylogenetic inferences of this particular subtype should be interpreted with caution. Nevertheless, their surprisingly high rate of clustering and the most recent median year of diagnosis for any of the major subtypes could indicate that our cohort has managed to capture a substantial segment of a network of HCV4d in HIV-positive MSM. A previous study has demonstrated that clusters of HCV1a in MSM in the Netherlands and Belgium have persisted despite unrestricted DAA therapy [58], which could be the case for HCV4d in MSM as well. Unfortunately, due to the age of our HCV4d samples which predate the policy changes regarding access to DAA in 2018 in Belgium, we are unable to investigate whether a similar dynamic is inherent to the HCV4d epidemic. Second, that no clusters with two or more cohort sequences contained control sequences is evidence of a disconnect between publicly available sequences and our cohort. This could be explained with the relative scarcity of HCV sequence data in general or the lack of good data with relevance to the epidemic in Belgium specifically. The abundant sequence data obtained here can serve as an important epidemiological resource going forward for researchers studying the dynamics of transmission domestically and abroad. Third, while our cohort is large and diverse and based on longer sequences than typically used, more focused studies can illuminate fragments of an epidemic at a higher resolution. In a 2019 cohort of PWID from Brussels, the prevalence of HCV was 41.1% [59]. While there are no good data on the incidence of HCV in HIV-positive MSM in Belgium, the neighbouring countries of the Netherlands and France suggested an incidence of 5.5 and 4.5/1000 person years of follow-up in 2016 and 2017 [60,61]. With similar frequencies of PWID and HIV-positive MSM in our four major subtypes (49/207 (23.67%) and 55/207 (26.57%)) we can expect the sampling densities of the two communities to be very different, and no direct comparison of the clustering rate between the two should be attempted. We therefore expect the true dynamics of HCV3a, which had the highest proportion of PWID in our combined cohort, to be much more active than what the evidence of this study suggests. On the other hand, the HIV-positive MSM specifically could be relatively well-represented in our cohort. In one recent study, 88% of HCV1a-infected HIV-positive MSM from Belgium and the Netherlands in two two-year periods were in transmission clusters [58], which is comparable to the 33/39 (84.61%) seen in our cohort. More data, preferably prospective sampling of PWID in Belgium, is needed for a more in-depth and updated analysis on this specific sub-population that makes up a majority of the contemporary acute HCV infections in Belgium. This is especially urgent if Belgium is to meet the WHO target of HCV elimination by 2030 [62], since the number of patients treated annually is expected to drop below the threshold of 1200 required to meet the WHO criteria, as a diminishing number of HCV infections will necessitate an upscaling of screening [63].

## 5. Conclusions

Our phylogenetic analysis of a large and diverse cohort in Flanders, Belgium suggests that the strains historically associated with iatrogenic infections have failed to gain a foothold in the communities associated with active transmission in present-day Belgium. Additionally, HCV1a and 4d appear to be actively transmitted across HIV-positive MSM populations attending clinical centres in different cities. Targeted research on the HCV3a in PWID in Belgium is necessary to characterize the dynamics of transmission of this key population specifically.

## Figures and Tables

**Table 1:** Rows contain information on the combined cohort and the three constituent cohorts of the participating clinical centres for sequences where ≥90% of the ORF was covered to a depth >30, and the subtype was confirmed using both BLAST and Genome Detective.

Cohort (# of ORFs)	Subtype	Gender	Gender of Attraction	HIV Status	IVDU History	Country of Origin	Iatrogenic Infections	Year of Birth (IQR)	Year of Diagnosis (IQR)	Year of Sampling (IQR)
Total (n = 224)	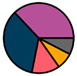	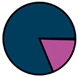	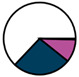	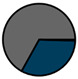	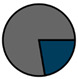	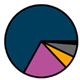	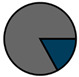	1959 (1966–1973)	2000 (2008–2012)	2013 (2015–2016)
Leuven (n = 111)	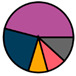	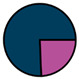	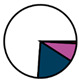	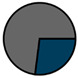	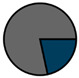	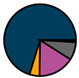	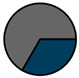	1955 (1964–1972)	1999 (2005–2012)	2014 (2015–2016)
Antwerp (n = 42)	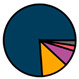	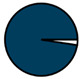	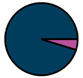	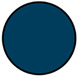	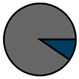	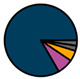	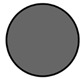	1964 (1967–1972)	2009 (2011–2013)	2009 (2011–2013)
Genk (n = 71)	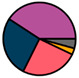	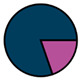	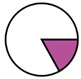	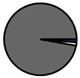	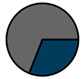	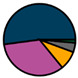	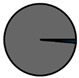	1960 (1966–1975)	2002 (2008–2012)	2013 (2015–2016)
Legend	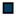 1a 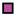 1b 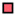 3a 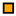 4d 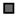 Other	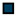 Male 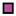 Female 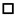 No data	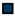 Same 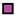 Opposite 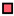 Either 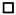 No data	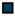 HIV+ 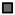 HIV- 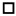 No data	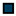 IVDU 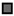 No recorded IVDU	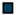 Belgium 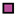 Europe 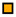 Russia and Asia 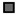 Other 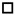 No data	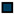 Iatrogenic 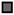 Not iatrogenic			

**Table 2:** Characteristics of the sequences committed to a phylogenetic analysis.

	1a (n = 78)	1b (n = 83)	3a (n = 29)	4d (n = 17)
Male	70 (89.74%)	57 (68.67%)	28 (96.55%)	15 (88.24%)
Year of birth (IQR)	1965 (1961–1971)	1961 (1950–1970)	1967 (1965–1979)	1968 (1966–1973)
Year of diagnosis (IQR)	2009 (2006–2012)	2004 (1998–2011)	2009 (2002–2013)	2011 (2010–2013)
Year of sampling (IQR)	2014 (2011–2015)	2015 (2014–2016)	2015 (2014–2017)	2013 (2011–2017)
IVDU	24 (30.77%)	6 (7.23%)	15 (51.72%)	4 (23.53%)
HIV + MSM	39 (50.00%)	3 (3.61%)	2 (6.90%)	13 (76.47%)
Iatrogenic infection	5 (6.41%)	24 (28.92%)	3 (10.34%)	1 (5.88%)
Migrant	13 (16.67%)	31 (37.35%)	13 (44.83%)	6 (35.29%)
Viral Load (IQR)	1.9 M (0.53 M–2.57 M)	1.2 M (0.44 M–1.90 M)	1.4 (0.43 M–1.90 M)	0.9 M (0.29 M–4.60 M)

**Table 3:** Transmission clusters of the four major subtypes in our cohort were defined in Cluster Picker using a constant bootstrap threshold of 90% with four genetic distances from 2 to 8 per subtype. Odds ratio and *p*-values were calculated using Bonferroni-corrected Fisher’s exact test with *p* < 0.0125 considered statistically significant.

Genetic Distance	No. of Clusters	In Cluster	IVDU Odds Ratio (*p*-Value)	HIV + MSM Odds Ratio (*p*-Value)	Iatrogenic Odds Ratio (*p*-Value)	Migrant Odds Ratio (*p*-Value)
1a						
2	9	28/78 (35.9%)	0.25 (*p* = 0.022)	37.00 (*p* = 0.000)	0.43 (*p* = 0.649)	0.27 (*p* = 0.119)
4	10	42/78 (53.85%)	0.63 (*p* = 0.461)	18.33 (*p* = 0.000)	0.20 (*p* = 0.175)	0.20 (*p* = 0.030)
6	13	49/78 (62.82%)	0.59 (*p* = 0.319)	10.88 (*p* = 0.000)	0.13 (*p* = 0.061)	0.12 (*p* = 0.003)
8	14	57/78 (73.08%)	1.15 (*p* = 1.000)	6.76 (*p* = 0.002)	0.08 (*p* = 0.017)	0.10 (*p* = 0.001)
			1b			
2	0	-	-	-	-	-
4	1	2/83 (2.41%)	0.00 (*p* = 1.000)	0.00 (*p* = 1.000)	0.00 (*p* = 1.000)	0.00 (*p* = 0.526)
6	3	6/83 (7.23%)	2.88 (*p* = 0.372)	0.00 (*p* = 1.000)	0.47 (*p* = 0.667)	0.83 (*p* = 1.000)
8	8	18/83 (21.69%)	1.91 (*p* = 0.606)	0.00 (*p* = 1.000)	1.80 (*p* = 0.379)	0.26 (*p* = 0.054)
3a						
2	0	-	-	-	-	-
4	0	-	-	-	-	-
6	1	3/29 (10.34%)	2.00 (*p* = 1.000)	0.00 (*p* = 1.000)	0.00 (*p* = 1.000)	2.73 (*p* = 0.573)
8	1	4/29 (13.79%)	0.92 (*p* = 1.000)	0.00 (*p* = 1.000)	0.00 (*p* = 1.000)	1.27 (*p* = 1.000)
4d						
2	4	9/17 (52.94%)	0.00 (*p* = 0.029)	4.80 (*p* = 0.294)	0.00 (*p* = 0.471)	0.08 (*p* = 0.050)
4	3	13/17 (76.47%)	0.18 (*p* = 0.219)	5.50 (*p* = 0.219)	0.00 (*p* = 0.235)	0.44 (*p* = 0.584)
6	4	15/17 (88.24%)	0.25 (*p* = 0.426)	inf (*p* = 0.044)	inf (*p* = 1.000)	inf (*p* = 0.515)
8	4	15/17 (88.24%)	0.25 (*p* = 0.426)	inf (*p* = 0.044)	inf (*p* = 1.000)	inf (*p* = 0.515)

## Data Availability

The 224 open reading frames of HCV obtained here, including the 207 sequences used in the generation of the phylogenies, are publicly available on GenBank (OR497849—OR498072). The clinical data on the patients in our cohort cannot be shared.

## Supplementary Materials

The following supporting information can be downloaded at: https://www.mdpi.com/article/10.3390/v15122391/s1; Figure S1: Maximum likelihood phylogeny overview of every cohort sequence qualifying for phylogenetic analysis; Figure S2: Phylogenetic tree of HCV1b; Figure S3: Phylogenetic tree of HCV3a.
