# Supplementary material for: Phylogenetic Analysis of Hepatitis C Virus Infections in a Large Belgian Cohort Using Next-Generation Sequencing of Full-Length Genomes
Source: Viruses. 2023 Dec 8;15(12):2391. doi: 10.3390/v15122391 (PMC10747466; doi:10.3390/v15122391)
Supplement: Supplementary file 1 [file viruses-15-02391-s001.zip › Supplementary_Materials.pdf]

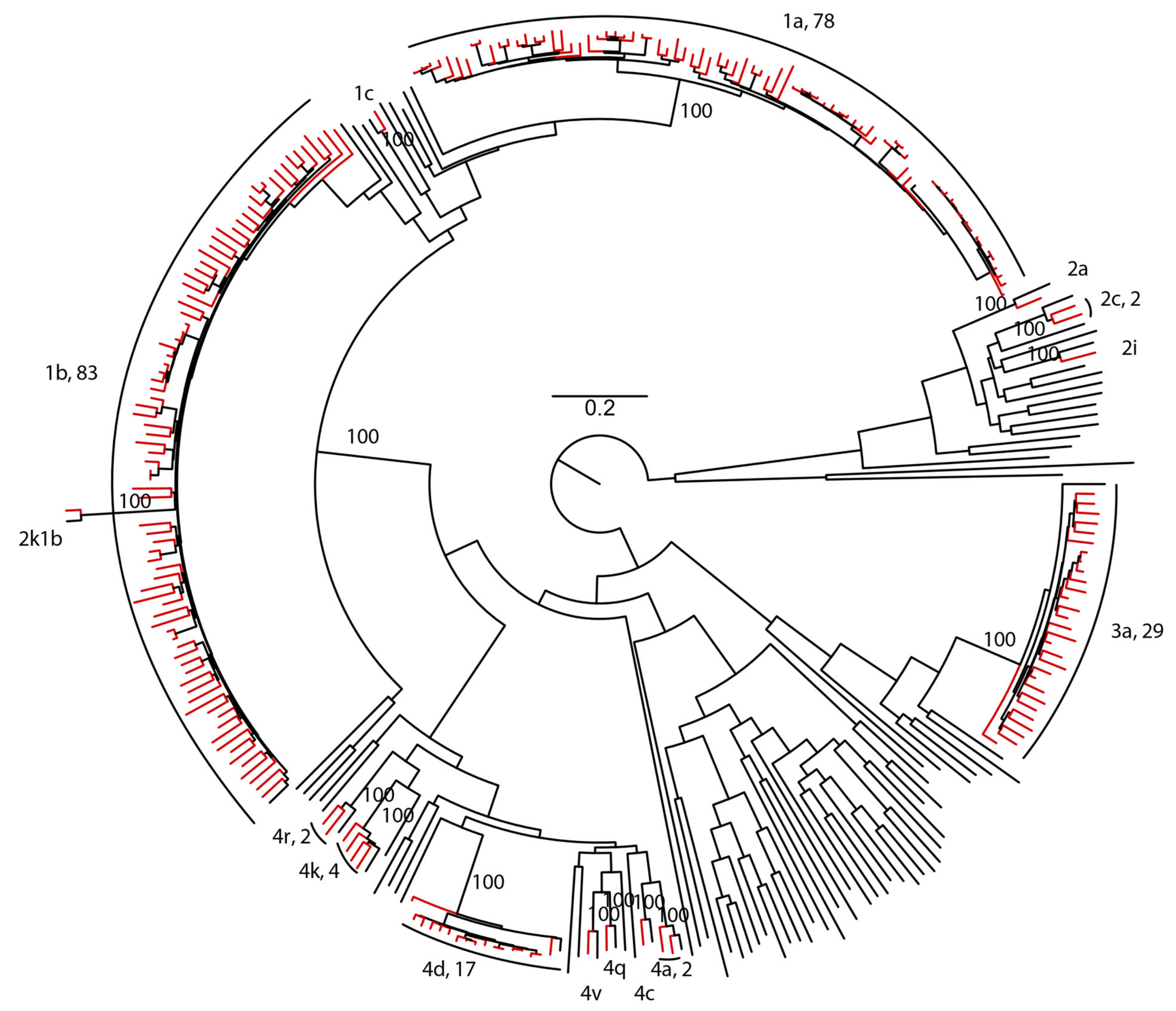

Supplementary Figure S1: Maximum likelihood phylogeny overview of every cohort sequence qualifying for phylogenetic analysis

Sequences were aligned using MAFFT and the tree made with IQ-TREE 2 with automatic substitution model (GTR+F+R10) and 1,000 ultra-fast bootstrap replicates. All 224 sequences of the cohort (red) with 90 references representing individual HCV subtypes from the International Committee for the Taxonomy of Viruses. The values on branches display the bootstrap support for clades of subtypes.

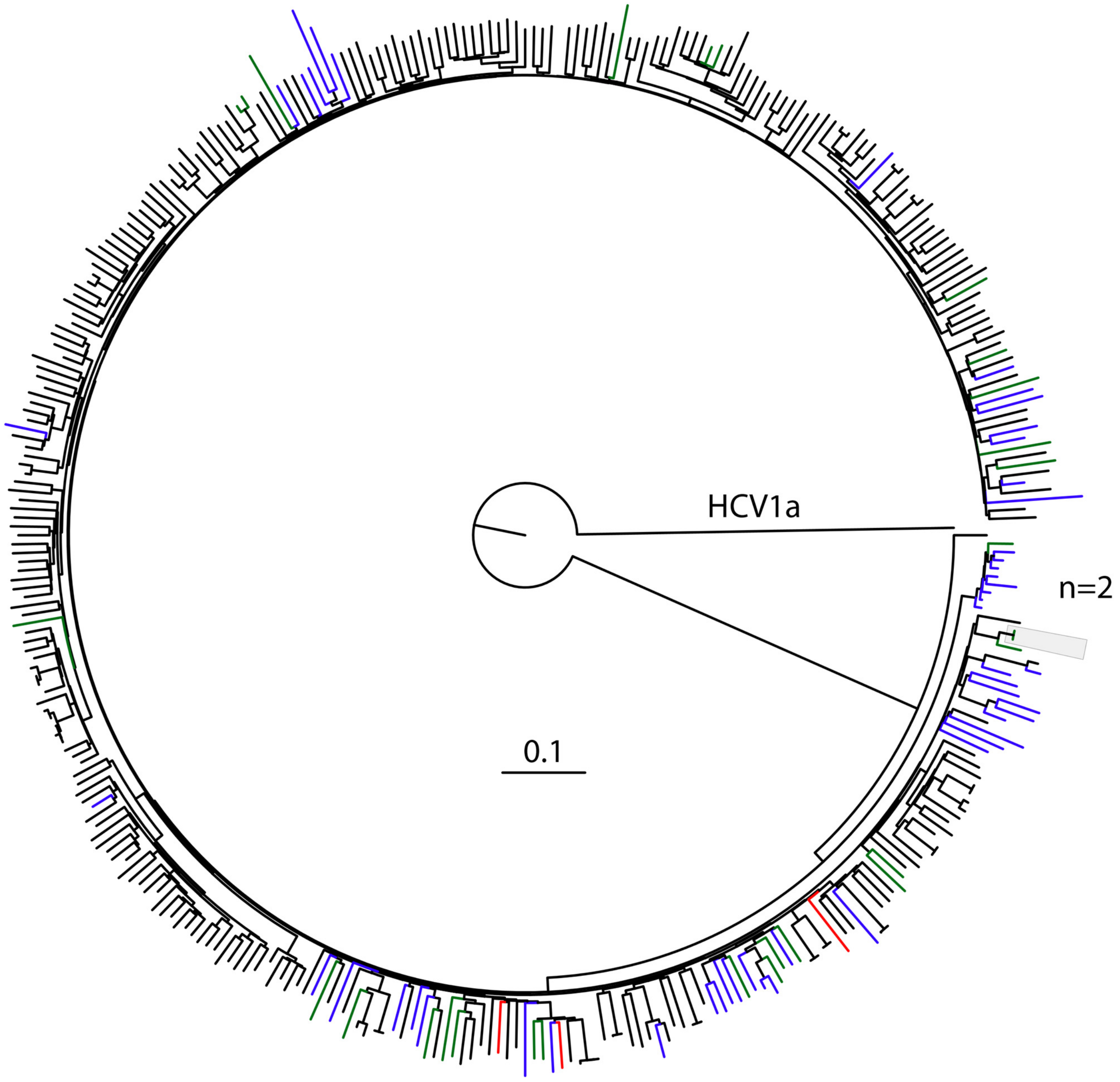

Supplementary Figure S2: Phylogenetic tree of HCV1b

A maximum-likelihood tree was constructed based on HCV1b ORFs using MAFFT and IQ-Tree 2 with automatic substitution model detection (TIM2+F+R10) and 1,000 ultra-fast bootstrap replicates. Blue, red, and green branches correspond to samples originating from Leuven, Antwerp, and Genk, respectively. Clusters based on bootstrap and genetic distance thresholds of 90% and 4 and are highlighted in grey. The two patients in the only cluster were not recorded as either MSM, HIV+, PWID, or with a potential iatrogenic infection.

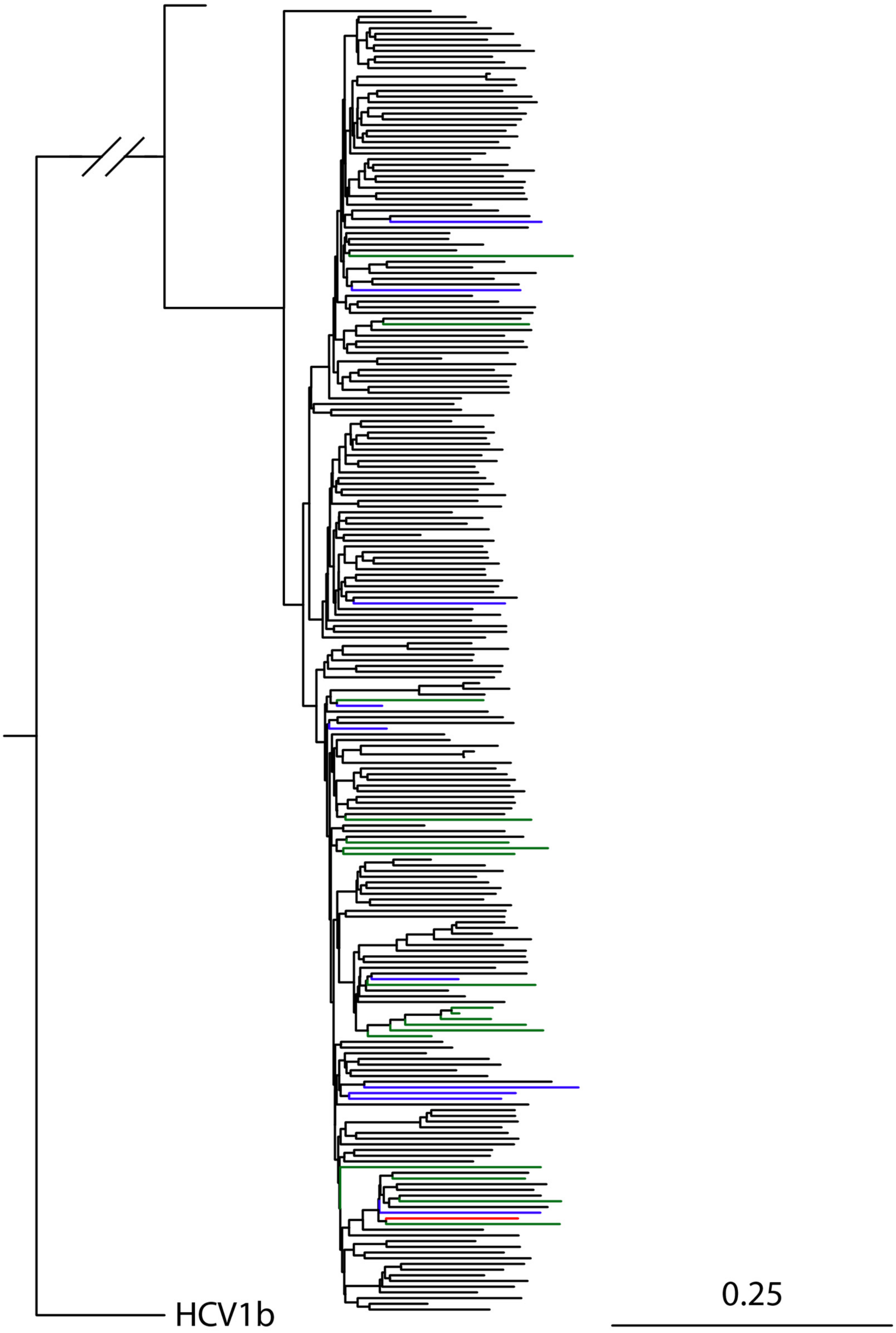

Supplementary Figure S3: Phylogenetic tree of HCV3a

A maximum-likelihood tree was constructed based on HCV3a ORFs using MAFFT and IQ-Tree 2 with automatic substitution model detection (GTR+F+R6) and 1,000 ultra-fast bootstrap replicates. Blue, red, and green branches correspond to samples originating from Leuven, Antwerp, and Genk, respectively. No clusters were identified using bootstrap and genetic distance thresholds of 90% and 4.
