# Supplementary figures and images for: Phylogenetic Analysis of Hepatitis C Virus Infections in a Large Belgian Cohort Using Next-Generation Sequencing of Full-Length Genomes
Source: Viruses. 2023 Dec 8;15(12):2391. doi: 10.3390/v15122391 (PMC10747466; doi:10.3390/v15122391)

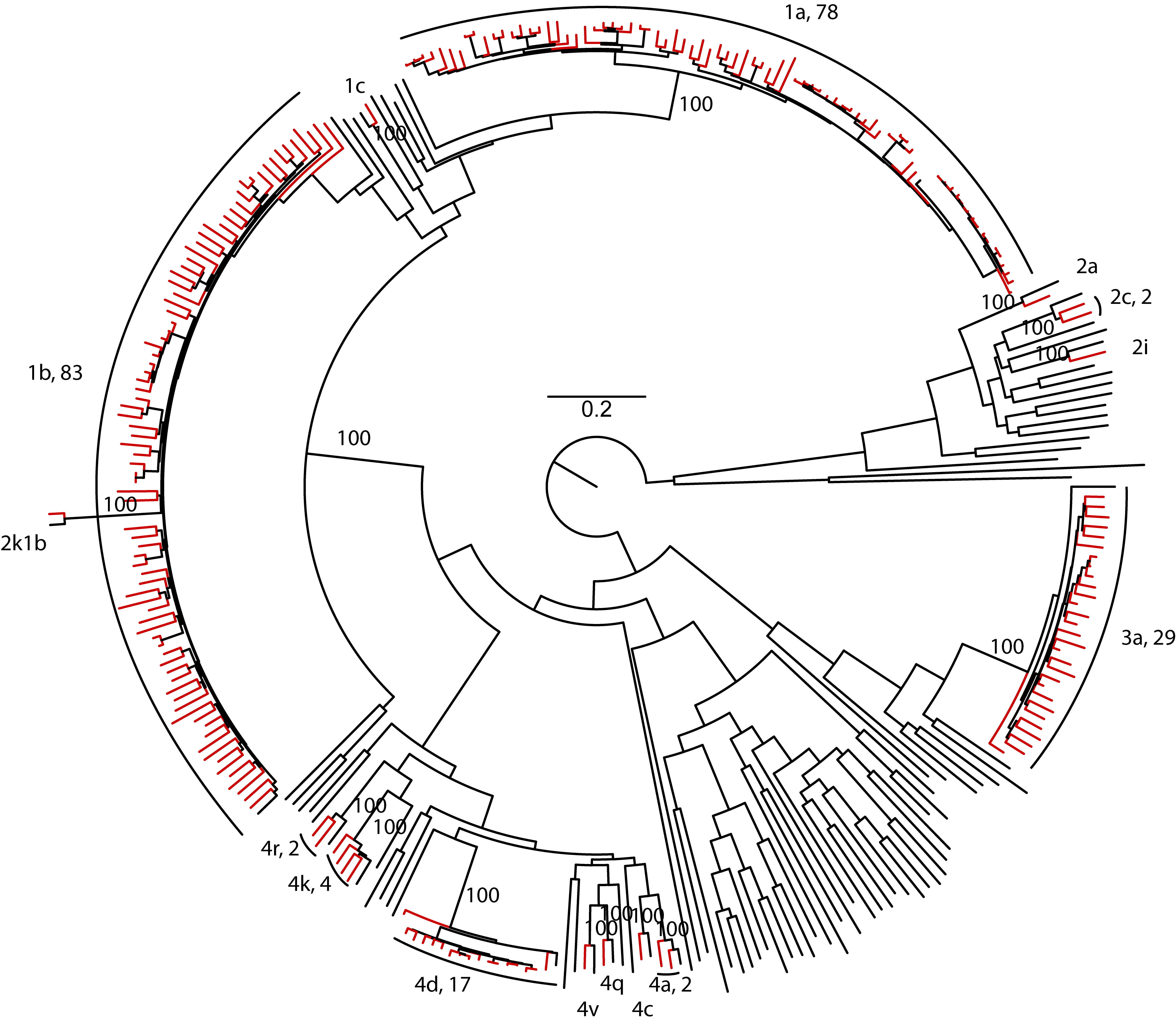

Supplement: Supplementary file 1 [file viruses-15-02391-s001.zip › FigureS1.tif]

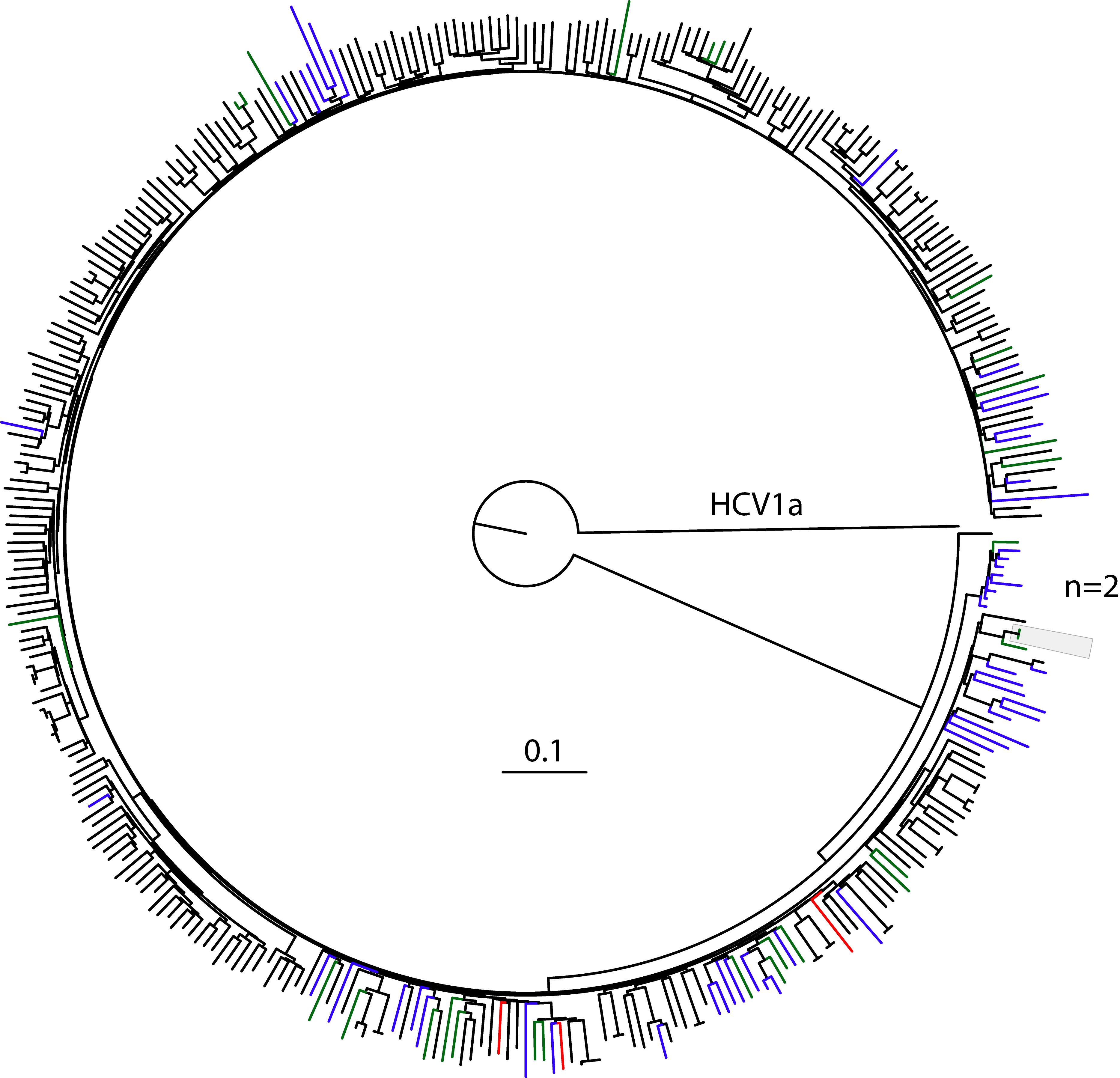

Supplement: Supplementary file 1 [file viruses-15-02391-s001.zip › FigureS2.tif]

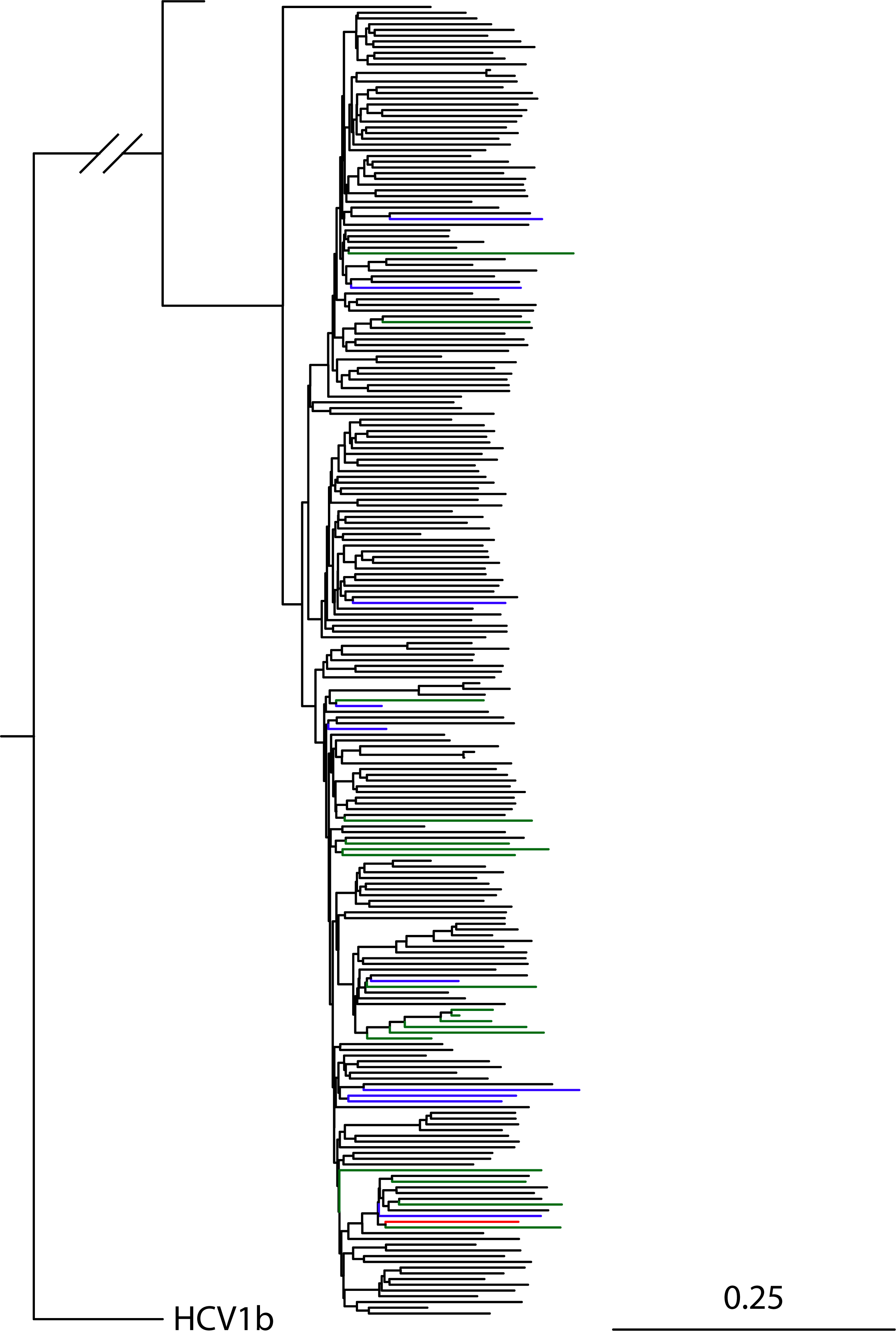

Supplement: Supplementary file 1 [file viruses-15-02391-s001.zip › FigureS3.tif]
